# Dietary Strategies in the Prevention of MASLD: A Comprehensive Review of Dietary Patterns Against Fatty Liver

**DOI:** 10.3390/metabo15080528

**Published:** 2025-08-04

**Authors:** Barbara Janota, Karolina Janion, Aneta Buzek, Ewa Janczewska

**Affiliations:** 1 Department of Basic Medical Sciences, Faculty of Public Health in Bytom, Medical University of Silesia in Katowice, Piekarska 18, 41-902 Bytom, Poland; 2 Silesian College of Medicine in Katowice, Adama Mickiewicza 29, 40-085 Katowice, Poland; karolinajanion@gmail.com; 3 Faculty of Public Health in Bytom, Medical University of Silesia in Katowice, Piekarska 18, 41-902 Bytom, Poland; aneta.buz@interia.pl; 4 ID Clinic Mysłowice, Janowska 19, 41-400 Mysłowice, Poland; e.janczewska@poczta.fm

**Keywords:** MASLD, metabolism, diet, nutrients

## Abstract

Understanding the components of the diet, food groups, and nutritional strategies that help prevent MASLD (Metabolic Dysfunction-Associated Steatotic Liver Disease) is essential for identifying dietary behaviors that can stop the progression of this condition, which currently affects over one-quarter of the global population. This review highlights the importance of including antioxidant nutrients in the diet, such as vitamins C and E, CoQ10, and polyphenolic compounds. It also emphasizes substances that support lipid metabolism, including choline, alpha-lipoic acid, and berberine. Among food groups, it is crucial to choose those that help prevent metabolic disturbances. Among carbohydrate-rich foods, vegetables, fruits, and high-fiber products are recommended. For protein sources, eggs, fish, and white meat are preferred. Among fat sources, plant oils and fatty fish are advised due to their content of omega-3 and omega-6 fatty acids. Various dietary strategies aimed at preventing MASLD should include elements of the Mediterranean diet or be personalized to provide anti-inflammatory compounds and substances that inhibit fat accumulation in liver cells. Other recommended dietary models include the DASH diet, the flexitarian diet, intermittent fasting, and diets that limit fructose and simple sugars. Additionally, supplementing the diet with spirulina or chlorella, berberine, probiotics, or omega-3 fatty acids, as well as drinking several cups of coffee per day, may be beneficial.

## 1. Introduction

A common characteristic of most metabolic disorders is their diet-dependency [1]. Daily dietary choices may exert a greater influence on the development of metabolic diseases than non-modifiable factors such as sex, age, or genetic predisposition [1,2]. One notable condition with a metabolic etiology is Metabolic Dysfunction-Associated Steatotic Liver Disease (MASLD), formerly known as Non-Alcoholic Fatty Liver Disease (NAFLD) [3]. The global prevalence of this condition is steadily increasing and is currently estimated at 38% worldwide and approximately 25% in Europe [4].

According to the 2023 diagnostic criteria established by the American Association for the Study of Liver Diseases (AASLD), the diagnosis of MASLD is based on imaging or histological evidence of hepatic steatosis, along with the presence of at least one of the following five metabolic risk factors: a body mass index (BMI) >25 kg/m^2^ or waist circumference ≥94 cm in men and ≥80 cm in women, blood pressure ≥130/85 mm Hg or ongoing antihypertensive treatment, serum triglyceride concentration ≥1.7 mmol/L (150 mg/dL) or pharmacological treatment for hypertriglyceridemia, high-density lipoprotein (HDL) cholesterol ≤1.0 mmol/L (40 mg/dL) in men and ≤1.3 mmol/L (50 mg/dL) in women or treatment for dyslipidemia, or fasting glucose ≥5.6 mmol/L (100 mg/dL) or a confirmed diagnosis of type 2 diabetes [5].

The pathophysiology of MASLD is closely associated with insulin resistance, accumulation of visceral adipose tissue, and elevated concentrations of pro-inflammatory mediators—all of which may result from chronically suboptimal dietary patterns [6]. A BMI above 25 kg/m^2^ or excessive waist circumference is indicative of overweight or obesity, or visceral fat accumulation, typically stemming from excessive caloric intake [6]. From a nutritional standpoint, arterial hypertension is often linked to chronic excessive sodium intake [7]. Hypertriglyceridemia is most commonly a result of high intake of simple carbohydrates, while reduced HDL cholesterol is frequently associated with excessive consumption of trans fatty acids [8,9]. Elevated blood glucose levels, when not attributable to pancreatic β-cell dysfunction, are typically the outcome of poor dietary composition and excessive consumption of refined carbohydrates [10].

Nutritional management is a critical component in the treatment of MASLD, as emphasized by hepatology societies in their dietary guidelines [11]. The significance of nutrition in hepatic steatosis is further underscored in the AASLD Practice Guidance on the Clinical Assessment and Management of Nonalcoholic Fatty Liver Disease, where dietary intervention is identified as a primary therapeutic strategy in the early stages of MASLD [5].

It has been demonstrated that dietary modifications aimed at preventing the accumulation of lipid droplets within hepatocytes can not only halt disease progression but also significantly reduce the degree of steatosis [11]. This is particularly relevant given the potential complications associated with MASLD, including the development of chronic hepatic inflammation, subsequent fibrosis, and progression to hepatocellular carcinoma [11].

After analyzing MASLD through dietary patterns—and considering both its etiological factors and clinical consequences—we conclude that a comprehensive, multifaceted approach to dietary prevention is warranted.

## 2. Methods

An analysis of dietary components, food groups, and overall nutritional strategies in the context of MASLD prevention appears necessary in order to identify dietary behaviors that support both metabolic health and liver function. This article aims to highlight beneficial dietary elements—ranging from individual nutrients to broader dietary patterns—that contribute to the prevention of MASLD.

To achieve this, a comprehensive review was conducted. The search was carried out in June 2025 using the electronic PubMed database. The review included publications in English. Both human and animal studies were presented, as well as in vitro experiments, all of which were clearly indicated in the text. Clinical studies and meta-analyses were accepted, whereas review articles comprised a minority of the literature presented. The following keywords were used to identify relevant scientific publications: MASLD AND diet—724 publications, MASLD AND nutrition—847 publications, MASLD AND Mediterranean Diet—57 publications, MASLD AND intermittent fasting—135 publications, MASLD AND anti-inflammatory dietary ingredients—38 publications, MASLD AND polyphenols—24 publications, MASLD AND omega-3—19 publications, fruits vegetables AND MASLD—11 publications.

A total of 109 articles were ultimately included in the final analysis. Of these, 105 were published between 2020 and 2025, while the remaining 4 were published prior to 2020. The last search was conducted on 20 July 2025.

The illustration of the AASLD Practice Guidance on the Clinical Assessment and Management of Nonalcoholic Fatty Liver Disease was prepared using the Canva online graphic design tool. Portions of the text were translated from Polish into English using the ChatGPT language model (OpenAI, 3180 18th StreetSan Francisco, CA 94110, USA, June 2025 version). The translation was subsequently manually edited by the authors.

## 3. Dietary Components in MASLD Prevention

### 3.1. Antioxidants

Among dietary components, some exhibit antioxidant properties, which may exert protective effects throughout the body, including via hepatocytes. This is a desirable action because, understanding MASLD in the context of overall metabolic disorders, attention should be paid to antioxidant interventions in the state of overweight and/or obesity, and in cardiological problems accompanied by increased concentration of interleukin-6 (IL-6), IL-1β, or tumor necrosis factor α (TNF-α), or an increased percentage of CD8 lymphocytes. MASLD itself is also characterized by increased expression of miR-125b, a microRNA that regulates inflammatory processes [12,13].

The most commonly known dietary antioxidants include vitamins C and E. Vitamin E, in particular, is frequently used therapeutically in metabolic dysfunction-associated steatohepatitis (MASH) due to its lipid-specific antioxidant effects [14,15].

#### 3.1.1. Vitamin E

Vitamin E refers to a group of chemical compounds, including tocotrienols and tocopherols, known for their anti-inflammatory effects. It can be reduced to its active form in the presence of other antioxidants such as vitamin C or ubiquinol [15]. Vitamin E is synthesized exclusively by plants, but due to its transfer through the food chain, it is also present in animal-derived products. Major dietary sources include plant oils, nuts, seeds, and sprouts [16,17,18]. In the context of hepatic steatosis, vitamin E has been shown to significantly reduce liver inflammation, as confirmed through histological examination in MASLD patients [17,19]. A meta-analysis of 24 studies evaluating the impact of vitamin E on liver histology and enzymes concluded that it improves liver function and reduces inflammation [18].

#### 3.1.2. Vitamin C

Vitamin C is a potent antioxidant that also exerts protective effects on the liver. It is present in many fruits and vegetables, particularly parsley, bell peppers, blackcurrants, and kiwis [16]. Studies on individuals with liver steatosis have demonstrated a correlation between serum vitamin C levels and the degree of liver fibrosis, as measured by elastography [19]. Lower levels of vitamin C were especially evident in overweight individuals [19]. In a cohort of 4494 Americans who underwent liver elastography, an inverse relationship was observed between vitamin C levels and MASLD severity, fibrosis progression, and cirrhosis stage [20].

#### 3.1.3. Coenzyme Q10

Ubiquinol, the reduced form of coenzyme Q10 (CoQ10), plays a role not only in electron transport in the respiratory chain but also in combating inflammation and regulating hepatic lipid metabolism, thereby preventing steatosis [21]. While primarily synthesized endogenously, CoQ10 can be obtained through diet, particularly from organ meats (e.g., liver, heart), red meat, and fatty fish. Dietary supplementation with CoQ10 is also common. In a study evaluating the effects of six months of daily 240 mg CoQ10 supplementation, a reduction in liver steatosis was observed via elastography, with a decrease from 304.8 ± 37.4 dB/m to 280.9 ± 33.4 dB/m [22]. Moreover, interventions aimed at increasing CoQ10 synthesis may contribute to MASLD prevention [23].

#### 3.1.4. Polyphenols

Polyphenols are plant metabolites characterized by their phenolic structures and potent antioxidant properties. They prevent fatty liver disease through antioxidant mechanisms. Polyphenols inhibit lipogenesis (fat synthesis in the liver) by affecting sterol regulatory element-binding protein-1c (SREBP-1c) and acetyl-CoA carboxylase (ACC) and increase β-oxidation of fatty acids (e.g., by activating peroxisome proliferator-activated receptor α (PPAR-α)).They help prevent low-density lipoprotein (LDL) cholesterol oxidation and are more abundant in plants than vitamins C and E, supporting their regular dietary inclusion [24].

• Resveratrol

Found predominantly in red grapes, especially the Pinot Noir variety, as well as in pomegranates and blackberries, resveratrol is well known for its cardiovascular protective effects [25]. A review of studies examining the impact of resveratrol on MASLD found insufficient evidence of a direct benefit in humans, though animal and in vitro studies have shown activation of firtuin1 (SIRT-1) and 5′AMP-activated protein kinase (AMPK) pathways, supporting homeostasis and promoting autophagy, along with antioxidant effects in hepatocytes [25]. While no direct effect on steatosis was confirmed in clinical trials, reduced inflammatory markers suggest potential indirect metabolic benefits [25].

• Quercetin

This polyphenol is abundant in many fruits and vegetables, particularly green leafy vegetables, tomatoes, and berries [25]. In vitro studies have shown that quercetin maintains high levels of glutathione, superoxide dismutase, and catalase, contributing to antioxidant defense [25,26]. Clinical studies in MASLD patients indicate that quercetin supplementation (500–1000 mg/day) may reduce alanine aminotransferase (ALT) and gamma-glutamyl transferase (GGT) levels, suggesting its potential therapeutic role [25].

• Curcumin

A polyphenolic compound responsible for the health benefits of turmeric, curcumin exhibits strong antioxidant properties. A meta-analysis showed that it helps prevent hepatic lipid accumulation and reduces aspartate aminotransferase (AST) levels and BMI in MASLD patients [27]. Researchers suggest curcumin as an adjunctive therapy for MASLD.

• Catechins

Epicatechin, found in green tea, has hepatoprotective properties. In vitro studies on MASLD-affected cells demonstrated positive effects on lipid metabolism via stimulation of PPARα and PPARγ [28]. Animal studies showed that epigallocatechin gallate (EGCG) reduced liver steatosis, supporting its anti-MASLD potential [29]. Additionally, epicatechin helps maintain normal glucose levels and has antifibrotic effects—important due to MASLD’s link to type 2 diabetes and progression to cirrhosis [30].

• Silymarin

Silymarin, a bioactive component of milk thistle oil, is one of the most widely studied hepatoprotective agents. It has anti-inflammatory and antifibrotic effects and reduces serum transaminase levels [31]. A review on silymarin in chronic liver disease suggested a daily dose of 420 mg for MASLD patients [31].

• Ellagic Acid

Found mainly in red fruits such as strawberries, raspberries, grapes, and pomegranates, ellagic acid is a potent antioxidant. In a mouse study using exosomes from pomegranate containing ellagic acid, improved gut barrier function, reduced inflammation, and normalized liver fibrosis markers were observed, supporting its antifibrotic potential [32].

• Ginger Polyphenols

Ginger contains anti-inflammatory and antioxidant polyphenols that support liver detoxification [33]. These compounds inhibit the production of proinflammatory cytokines (e.g., TNF-α, IL-6) and downregulate nuclear factor kappa-light-chain-enhancer of activated B cells (NF-κB) and enzymes like cyclooxygenase-2 (COX-2) and inducible nitric oxide synthase (iNOS) [33]. Ginger supplementation has also shown lipid-lowering effects and improved HDL levels, with clinical studies reporting reduced liver enzyme levels, inflammation, and steatosis in MASLD patients [34].

### 3.2. Substances That Support Lipid Metabolism

In addition to components with primarily antioxidant effects, there are also compounds that mainly regulate metabolic processes. In MASLD, it is important to increase β-oxidation of fatty acids, improve the lipid export, and inhibit de novo lipogenesis and lipotoxicity. The ingredients discussed below actively support the presented processes.

#### 3.2.1. Choline

Choline is a chemical compound considered by some researchers to be a vitamin B4, although it is largely synthesized endogenously [16]. Its primary dietary sources include egg yolks and, to a lesser extent, organ meats, muscle meat, and milk [16]. In cases of choline deficiency, phospholipid synthesis is impaired, leading to fat accumulation in the liver due to disturbed VLDL cholesterol secretion and β-oxidation processes [35]. A study evaluating the relationship between choline intake and hepatic steatosis among Americans—based on dietary interviews and elastographic liver assessment—demonstrated that lower dietary choline intake was negatively correlated with liver fat content [36]. Another study on postmenopausal women with MASLD found that insufficient choline intake was associated with more advanced liver fibrosis [37].

#### 3.2.2. Alpha-Lipoic Acid

Alpha-lipoic acid is a potent antioxidant found mainly in organ meats (especially liver and heart), as well as in spinach and broccoli. It plays a key role in energy metabolism, lipid protection against oxidation, and regenerating other antioxidants (vitamins C and E, glutathione), thereby supporting anti-inflammatory processes. It also inhibits COX-2 expression, a proinflammatory enzyme [38,39]. In a rat model of MASLD induced by a high-fat diet, alpha-lipoic acid supplementation reduced liver inflammation, suggesting its protective role [39]. In a human study combining supplementation with silymarin and alpha-lipoic acid and adherence to a Mediterranean diet, improvements were observed in liver regeneration, reduced steatosis, and decreased metabolic disturbance markers such as waist circumference and visceral fat content [40].

#### 3.2.3. Betaine

Betaine, a derivative of amino acids, is found naturally in sugar beets and sprouted grains and is primarily derived from cereal products, though it is increasingly included in dietary supplements [41]. It is produced endogenously as part of choline metabolism [41]. Plasma betaine levels have been shown to be lower in individuals with MASLD compared to healthy controls [41]. A review of betaine’s effects on the gut–liver axis emphasizes its role in maintaining intestinal barrier integrity, which is crucial for preventing MASLD progression [41]. Though its precise mechanism is unclear, betaine’s lipotropic and insulin-sensitizing effects have been well documented, and it is now considered a promising therapeutic agent in MASLD management [42,43].

#### 3.2.4. Berberine

Berberine is a plant-derived compound from the barberry shrub, traditionally used for centuries to manage lipid and carbohydrate metabolism disorders [44]. Its mechanism against liver steatosis involves activation of the enzyme, promoting fatty acid oxidation in hepatocytes [44]. Berberine also enhances intestinal barrier integrity, thereby reducing hepatic exposure to toxins [45]. In an animal study of induced MASLD, berberine supplementation was found to reduce disease severity, lower inflammation, and improve insulin sensitivity [46].

### 3.3. Fermented Products and Probiotics

Metabolic disturbances in MASLD are associated with gut microbiota dysbiosis and impaired intestinal barrier function, highlighting the importance of the gut–liver axis in MASLD pathogenesis [47]. The liver, as the first organ exposed to gastrointestinal substances via portal circulation, is particularly vulnerable to toxic bacterial metabolites [48]. Probiotics can reduce pathogenic bacteria by neutralizing endotoxins, restore microbiota balance, and limit harmful metabolite translocation to the liver [47]. Additionally, fermentation by probiotics generates short-chain fatty acids that modulate inflammation and gene expression, potentially slowing liver damage progression [49].

Probiotics occur naturally in many fermented products, including dairy products, fermented vegetables and fruits, cured sausages, sourdough bread, sauerkraut, and beverages such as beer and wine [50]. Probiotics in dairy products may inhibit MASLD progression by modulating Toll-like receptor 4 (TLR4) by lipopolysaccharide (LPS) signaling pathways in hepatocytes [51]. A clinical study in Iran found that regular consumption of low-fat milk improved liver markers, reduced insulin resistance, and lowered inflammation in MASLD patients [51]. Frequent yogurt intake (four or more times per week) was associated with a lower risk of MASLD [52].

Fermented vegetables and soy-based products possess anti-inflammatory properties and may play a supportive role in the prevention or management of chronic inflammatory diseases [53]. Laboratory studies show that kimchi components (fermented napa cabbage) reduce liver fat accumulation in both cell cultures and mouse models [54]. Further analyses confirm that kimchi inhibits the expression of genes involved in endoplasmic reticulum stress, lipogenesis, inflammation, and triglyceride accumulation in both in vitro and animal models [55].

Kombucha, a non-alcoholic fermented tea, is classified as a functional beverage due to its health-promoting properties [56]. Animal studies have shown that kombucha consumption improves liver and metabolic function, reduces hepatocyte apoptosis and fibrosis, and lowers ALT, AST, and triglyceride levels, indicating its potential in MASH prevention and the broader spectrum of fatty liver diseases [56].

### 3.4. Beta-Glucans

Beta-glucans are insoluble prebiotic polysaccharides found in dietary fiber as well as in yeast [57]. A review of beta-glucans’ effects on MASLD revealed animal studies indicating their hepatoprotective, anti-inflammatory, and antisteatotic properties [57]. In one study using oat-derived beta-glucan, researchers observed suppression of macrophage infiltration and inhibition of innate immune responses mediated by pattern recognition receptors (PRRs), effectively preventing fibrosis [58]. Although human trials are lacking, existing data on fiber and its fractions support increasing beta-glucan intake for liver health.

### 3.5. Spirulina and Chlorella

The microalgae *Arthrospira platensis* (spirulina) and *Chlorella vulgaris* (chlorella) are considered functional foods and have recently gained popularity, particularly in supplement form [59]. These algae are rich in bioactive compounds, vitamins, and minerals, with widely documented health benefits [59,60,61]. In mice with diet-induced MASLD, spirulina supplementation improved liver condition and reduced inflammation [60]. A meta-analysis reviewing studies on chlorella supplementation found it significantly lowered AST levels [61]. In a rat study, chlorella improved insulin sensitivity and reduced inflammation by downregulating gene expression in the p38 AMP-activated protein kinase (p38 AMPK)/TNF-α/NF-κB pathway [62].

### 3.6. Coffee

Coffee is attributed with multiple health benefits, owing to its content of caffeine, chlorogenic acids, and polyphenols, which exhibit anti-inflammatory and free radical-scavenging properties [63]. Chlorogenic acids activate the Nrf2 signaling pathway, promoting antioxidant defenses and suppressing inflammatory responses via inhibition of the toll-like receptor 4 (TLR4)/NF-κB pathway [64]. Coffee also supports lipid metabolism regulation [63].

A review of studies on coffee consumption and MASLD reduction suggests that in high-risk populations—such as individuals with obesity or diabetes—coffee intake may prevent hepatic steatosis [65]. Animal and in vitro studies involving THLE-2 hepatocyte lines indicate that daily caffeine consumption may alleviate MASH-related symptoms [66]. In a long-term study (over 11 years) involving 6592 participants with and without MASLD, researchers assessed the impact of coffee intake on MASLD risk. While no significant association was found in the overall analysis, a higher number of coffee cups consumed (≥2 per day) among MASLD patients was associated with a lower risk of fibrosis progression [67].

A summary of dietary components with preventive effects in MASLD is presented in Table 1.

## 4. Food Groups in MASLD Prevention

### 4.1. Carbohydrate-Rich Food Products

Given the predominant role of carbohydrates as an energy source in the diet, it is important to identify those particularly beneficial to health. Available data suggest that carbohydrates should provide around 50% of the daily energy intake. It is recommended to avoid refined carbohydrates in favor of unprocessed ones, as this may help reduce the risk of developing metabolic disorders. Meals should be based on products with a low glycemic index and glycemic load to support proper insulin balance. The diet should include at least 25–30 g of dietary fiber per day, while sugar intake should not exceed 5–10% of total daily energy. Such dietary choices may improve insulin sensitivity, including in the liver, and help reduce visceral inflammation [68].

For researchers studying the prevention of MASLD, maintaining a high antioxidant potential through the intake of vitamins A, E, C, zinc, selenium, and manganese is crucial—achievable largely through the consumption of optimal amounts of fruits and vegetables [69,70]. A cross-sectional study among MASLD patients showed that vegetable intake and regular physical activity had a protective effect against liver fibrosis and inflammation [71].

Beyond fruits and vegetables, whole-grain carbohydrate products are widely discussed in the literature as beneficial in MASLD prevention and diet therapy [72,73,74]. In a study assessing the impact of carbohydrate type on MASLD development in 255 women, fiber-rich carbohydrates with a low glycemic index were found to be protective [72]. Higher whole-grain intake was associated with a reduced risk of MASLD in a study involving 228 individuals evaluating healthy eating habits [74].

### 4.2. Protein-Rich Food Products

Daily intake of an optimal amount of protein, particularly essential amino acids, is necessary for tissue-building processes [16]. Protein sources include meat, fish, eggs, dairy, and legumes [16]. Researchers recommend choosing white meat and reducing red meat consumption to lower cardiometabolic risk [75,76]. Red meat is considered a risk factor for MASLD development [77]. Some evidence shows no clear link between white meat consumption and MASLD risk, supporting its use as a safer amino acid source [75].

Fish consumption is highly recommended for MASLD prevention, not only for its high-quality protein but also for its content of polyunsaturated fatty acids [16,77]. A study comparing a fish-only diet (with fish from freshwater sources) to a mixed meat–fish diet found that the fish-based diet reduced liver steatosis [78].

Egg intake is also analyzed in the MASLD context, not only for its amino acid content but also because eggs are a primary source of choline [79]. While no strong link was observed between egg consumption and liver steatosis risk, higher dietary choline intake was protective [80]. In an animal model of hepatic steatosis, a diet including hydrolyzed egg protein reduced fat accumulation in the liver [81].

Dairy products, in addition to providing essential amino acids, are also a source of calcium and vitamins (including A, D3, and B12). This group includes milk, cottage cheese, yogurt, hard and soft cheeses, kefir, and buttermilk. It has been shown that the consumption of low-fat dairy is associated with a reduced risk of cardiovascular diseases and the development of metabolic syndrome [82]. Moreover, these products have been demonstrated to positively influence cellular sensitivity to insulin.

Due to ongoing uncertainties regarding the relationship between the amount and type of dairy consumed and the risk of developing MASLD, Tirosh et al. conducted a 7-year cohort study assessing the association between dairy intake and MASLD development in a group of 316 individuals. The study found that the consumption of dairy products with low or moderate fat content was associated with a lower risk of MASLD, suggesting that the inclusion of appropriate dairy products in the diet may help protect against liver steatosis [82].

Legumes are a plant-based alternative to animal protein. Due to growing interest in plant-based diets, European researchers compared MASLD risk among consumers of plant vs. animal protein. In a study spanning over 10 years with more than 100,000 participants, replacing meat with legumes once a week slightly reduced MASLD risk, although further research is warranted [83].

### 4.3. Fat-Rich Food Products

MASLD prevention largely relies on promoting unsaturated fats while limiting saturated fats, especially those in trans configuration [84]. The intake of polyunsaturated fatty acids (PUFAs) and olive oil (a source of omega-9) has proven protective effects [85].

PUFAs are categorized into omega-3 and omega-6. Both are essential and must be obtained through diet or supplementation. Omega-6 is found in plant oils, while omega-3 is abundant in flaxseed and rapeseed oil, walnuts, fatty marine fish (herring, trout, mackerel), and marine algae [16]. Omega-3 and omega-6 fatty acids improve lipid profiles by lowering triglycerides and LDL cholesterol and raising HDL cholesterol—beneficial in the context of liver steatosis. Omega-3 metabolites also have strong anti-inflammatory effects and enhance insulin sensitivity [16,86]. Inflammation reduction is linked to decreased CRP, IL-6, TNF-α, intercellular adhesion molecule -1 (ICAM-1), and vascular cell adhesion protein 1 (VCAM-1) levels [87]. A clinical trial administering 503 mg DHA and 102 mg EPA for six months reduced alkaline phosphatase and liver fibrosis [88].

A study comparing plasma omega-3 and omega-6 levels found lower omega-6 levels in MASLD patients, suggesting the need for a variety of plant oils in the diet, not just omega-3-rich sources [89]. A cohort study of over half a million people confirmed that omega-3 supplementation protects against liver disease—especially among women [90].

### 4.4. Ultra-Processed Foods

Ultra-processed foods refer to all food products that are industrially produced, contain food additives, are attractively packaged, and are prepared for immediate consumption. This category includes products such as chips, pretzels, crackers, cookies, processed dairy products, ready-to-eat meals, and colorful beverages. Consumption of these products is particularly discouraged in the need for prevention and treatment of MASLD. The consumption of such foods contributes to the development of metabolic diseases, including MASLD and its progression to inflammatory liver disease. This is primarily due to their high content of simple sugars and saturated fats, including trans fats, which promote the accumulation of lipids in hepatocytes [91,92].

A study analyzing dietary behaviors among 16,703 Australians over the age of 70 indicated that higher intake of highly processed foods was associated with an increased risk of MASLD. This finding underscores the importance of consuming minimally processed diets, even in older age [91]. Similarly, in a study conducted by Sun et al. involving 2458 women, it was demonstrated that the consumption of highly processed foods was associated with an increase in controlled attenuation parameter, further confirming their role in promoting the development of MASLD [92].

### 4.5. Alcohol

Alcohol is a substance whose consumption leads to or exacerbates liver steatosis. The intake of alcoholic beverages is discouraged in the prevention and treatment of MASLD. According to the AASLD guidelines, even moderate daily alcohol consumption—defined as 21–39 g for women and 31–59 g for men—can promote hepatic inflammation and fibrosis, with particularly harmful effects in individuals with metabolic disorders such as obesity and diabetes [93].

Importantly, even lean individuals who consume alcohol are predisposed to developing MASLD, due to alcohol’s detrimental impact on gut microbiota. The role of gut microbiota in the development of MASLD is discussed in Section 3.3 [93].

## 5. Dietary Patterns in MASLD Prevention

### 5.1. Mediterranean Diet

The Mediterranean diet (MedDiet) emphasizes plant-based products, such as vegetables, fruits, whole grains, legumes, and nuts. It features a high intake of monounsaturated fats, mainly from olive oil, and moderate consumption of fish and fermented dairy, while limiting red meat and processed foods, including sweets [94].

According to current guidelines, the MedDiet is the first-line non-pharmacological intervention for MASLD due to its documented benefits in improving insulin sensitivity, reducing visceral fat, and enhancing liver biochemistry [11].

Its protective effect involves multiple mechanisms: limiting de novo lipogenesis, enhancing β-oxidation, exerting anti-inflammatory and antioxidant effects, regulating genes involved in lipid and glucose metabolism, and promoting gut microbiota health—partly through increased fiber intake [11,95].

Polyphenols and omega-3 fatty acids from fish also play a key role, modulating transcription factors such as SREBP-1c and PPAR-α/γ, with anti-inflammatory and antifibrotic properties [96].

Randomized trials, observational studies, and meta-analyses confirm that high adherence to the MedDiet is associated with lower MASLD risk and improved metabolic and anthropometric parameters in diagnosed patients [95,97]. For example, a meta-analysis by Del Bo’ et al. involving 10 studies and 737 adults showed a significant reduction in total cholesterol (mean difference: −0.46 mg/dL; *p* = 0.001). A trend toward reduced liver stiffness (mean difference: −0.42 kPa; *p* = 0.10) was also noted [98].

Notably, benefits were seen in non-Mediterranean populations, highlighting the MedDiet’s adaptability across cultural and dietary contexts. Health improvements may occur with or without weight loss. Sualeheen et al. observed improvements in liver enzymes (ALT, AST, GGT) even without significant body weight changes [99].

In summary, the MedDiet, due to its safety, palatability, and scientifically supported mechanisms, is a valuable element of MASLD prevention and treatment strategies. Its effectiveness, however, may vary depending on individual factors like lifestyle, physical activity, and long-term dietary adherence.

### 5.2. DASH Diet

Initially developed for blood pressure management, the DASH (Dietary Approaches to Stop Hypertension) diet is also effective in addressing metabolic disorders, including MASLD. It shares similarities with the MedDiet but places greater emphasis on sodium restriction. Unlike the Western diet, DASH promotes high intake of fiber, antioxidants, and minerals such as potassium, calcium, and magnesium—supporting metabolic function and anti-inflammatory action [74,94].

Observational studies show that DASH adherence is linked to lower risk of metabolic syndrome, insulin resistance, and improved insulin sensitivity—key factors in MASLD risk [74]. In overweight and obese individuals, higher DASH adherence correlated with lower triglycerides, fasting glucose, HOMA-IR, and liver fat content measured via MRI [100]. In a study by Xiao et al. involving over 3000 adults aged 40–75, the highest DASH adherence group had a 31% lower MASLD risk compared to the lowest adherence group (OR = 0.69; 95% CI: 0.55–0.86), partially explained by better BMI, HOMA-IR, triglyceride, and CRP levels [97].

DASH mechanisms include reducing chronic inflammation and oxidative stress while improving glucose and lipid metabolism. It also supports gut microbiota composition and has antihypertensive and hypolipidemic effects, further limiting MASLD progression to MASH [96,101]. Like MedDiet, DASH is safe, universal, and clinically effective for both prevention and management of MASLD.

### 5.3. High-Fiber Diet

A diet rich in dietary fiber is a key tool in managing MASLD. Soluble fiber fractions (e.g., beta-glucans, pectins, inulin) exert anti-inflammatory, lipid-lowering, and glucose-modulating effects, and positively influence gut microbiota—factors directly linked to MASLD pathogenesis [94,102]. Fiber sources include whole grains, vegetables, fruits, legumes, and nuts. Fiber fermentation by gut microbiota produces short-chain fatty acids (SCFAs) like butyrate, propionate, and acetate. These SCFAs positively affect gut barrier integrity, reduce inflammation, and modulate hepatic lipid metabolism via the gut–liver axis [95].

Clinical and epidemiological studies show inverse associations between fiber intake and MASLD risk. Intake of ≥25–30 g/day is linked to lower ALT and AST levels, reduced hepatic fat accumulation, and improved insulin resistance—regardless of weight loss [101,102].

Fiber-rich diets also lower triglycerides and LDL cholesterol while raising HDL cholesterol—important for MASLD patients with common dyslipidemias [95]. High fiber intake correlates with reduced risk of visceral obesity, metabolic syndrome, and type 2 diabetes. Studies confirm that whole-grain, fruit, and vegetable intake improves insulin sensitivity and reduces inflammatory markers (CRP, IL-6, TNF-α) [95].

Intervention studies show that high-fiber diets improve anthropometric measures (weight, waist circumference) and reduce the Fatty Liver Index (FLI). These effects may result from altered fat absorption, gut transit time, and postprandial glycemic response [94,102]. From a clinical perspective, increasing fiber intake in MASLD patients is a safe, well-tolerated, and broadly applicable intervention that may slow disease progression and improve overall metabolic health.

### 5.4. Flexitarian Diet

The flexitarian diet, based mainly on plant-derived foods with occasional animal product consumption, is emerging as an effective strategy for MASLD prevention and treatment. It emphasizes vegetables, fruits, legumes, nuts, seeds, and whole grains while limiting red meat and processed foods—factors linked to improved metabolic parameters and reduced disease risk [94,103]. A review by Castelnuovo et al. reported that diets with infrequent animal product consumption—including flexitarian diets—are associated with better metabolic profiles and lower MASLD prevalence [103]. Plant-based eaters also showed reduced visceral fat and lower liver enzyme levels (ALT, GGT) [104].

The fiber content in flexitarian diets, especially from legumes and whole grains, contributes to SCFA production, supporting anti-inflammatory action, gut barrier integrity, and reduced hepatic lipogenesis [104]. According to Quesada-Vázquez et al., plant-based diets promote beneficial bacteria such as *Faecalibacterium prausnitzii* and *Bifidobacterium*, positively impacting the gut–liver axis [105].

Compared to strict vegetarian or vegan diets, flexitarianism shows better long-term adherence and broader acceptability, making it more practical for MASLD management [104]. While randomized trials using imaging to assess hepatic steatosis are lacking, observational and review data support its efficacy in improving key metabolic parameters associated with MASLD [103,104].

### 5.5. Low-Fructose and Low-Sugar Diet

High fructose intake—especially from processed foods (e.g., sweetened beverages, baked goods, HFCS-containing products)—is a well-documented MASLD risk factor [95,106]. Unlike glucose, fructose is metabolized almost exclusively in hepatocytes, bypassing insulin regulation. It directly stimulates de novo lipogenesis, promoting triglyceride accumulation in the liver [106].

This pathway also increases uric acid production and oxidative stress, which contribute to mitochondrial damage, insulin resistance, and metabolic instability [107]. Observational and interventional studies link excessive fructose intake with adverse metabolic changes in both adults and children. Higher fructose intake, especially from non-fruit sources, is associated with increased visceral fat, hyperlipidemia, and inflammation [106,107].

In a study by Faienza et al. involving children with obesity, high fructose intake (excluding fruit) was linked to higher MASLD risk and elevated ALT, triglycerides, and HOMA-IR [107]. Fructose overconsumption also increases lipogenic gene expression (e.g., SREBP-1c) and impairs hepatic insulin signaling, promoting steatosis even without caloric excess [95,96].

Current guidelines recommend limiting fructose to <5–10% of daily energy intake. Eliminating sweetened beverages and HFCS products in favor of fresh fruits—which also provide fiber, vitamins, and anti-inflammatory polyphenols—is encouraged [95,101]. This strategy, within a balanced, minimally processed diet, is a valuable approach to preventing MASLD and its progression to MASH [98,107].

### 5.6. Intermittent Fasting (IF)

Intermittent fasting (IF) involves cyclic food intake restriction within designated time windows while maintaining overall caloric adequacy. Common IF models include alternate-day fasting (ADF), the 5:2 model (two low-calorie days per week), and time-restricted eating (TRE), typically 6–10 h per day. Benefits are attributed to enhanced autophagy, improved insulin sensitivity, and reduced oxidative stress [94,95].

In MASLD, IF may counter key pathophysiological mechanisms such as de novo lipogenesis, insulin resistance, chronic inflammation, and oxidative stress. Periodic food restriction lowers insulin levels, activates AMPK, enhances β-oxidation, and reduces hepatic fat accumulation [96].

In a 12-week study by Kord-Varkaneh et al., a TRE protocol (16:8) combined with a low-sugar diet resulted in weight loss, reduced body fat and liver steatosis (via CAP), and improved biochemistry (ALT, AST, GGT, triglycerides, LDL). Inflammatory markers (hs-CRP, cytokeratin-18) also declined [108].

IF downregulates proinflammatory cytokines (TNF-α, IL-6) and induces autophagy, protecting hepatocytes from damage and fibrosis [95,96]. It may also modulate clock gene expression, optimizing circadian glucose and lipid metabolism [95,96]. Meta-analyses confirm that IF improves body weight, fasting glucose, HOMA-IR, and ALT levels in overweight and obese individuals [101].

Despite its promise, IF is not suitable for all patients. Caution is advised in older adults, diabetics, or those requiring regular meals and medications. Long-term safety and efficacy in MASLD remain under investigation [94,100,101]. When appropriately tailored, IF can be a valuable non-pharmacological intervention in MASLD treatment.

### 5.7. Ketogenic Diet

The ketogenic diet (KD) is a high-fat (70–80%), low-carbohydrate (≤10% energy), moderate-protein (10–15%) diet aimed at inducing ketosis. In this state, ketone bodies (β-hydroxybutyrate, acetoacetate) become the primary energy source via enhanced β-oxidation in the liver [109].

In MASLD, the KD has gained attention for its potential to reduce hepatic fat, improve insulin sensitivity, and favorably affect lipid profiles and inflammation. Limiting carbohydrate—especially refined sugar—intake suppresses de novo lipogenesis and reduces liver triglyceride accumulation [95,101].

In a randomized trial by Chirapongsathorn et al., an 8-week KD led to greater reductions in weight, waist circumference, visceral fat, triglycerides, and AST compared to the DASH diet. Although liver fat reduction (via elastography) was not statistically significant, metabolic improvements were notable [109].

KD mechanisms include inhibition of de novo lipogenesis by reducing acetyl-CoA and malonyl-CoA availability, enhanced β-oxidation and ketogenesis, improved insulin sensitivity, lower postprandial glycemia, visceral fat loss, and reduced oxidative stress and inflammation [101,109].

Despite promising results, the KD’s long-term safety and efficacy in MASLD are debated. Concerns include increased LDL cholesterol and apolipoprotein B, especially with high saturated fat intake. Prolonged KD may also cause nutrient deficiencies, kidney stones, or constipation [109].

The KD may be an effective short-term tool for liver fat reduction and metabolic improvement in MASLD patients. Its implementation should be guided by thorough cardiometabolic risk assessment and supervised by healthcare professionals.

## 6. Conclusions

The dietary components, food groups, and nutritional strategies discussed above clearly highlight the essential role of diet and eating behaviors in both the prevention and management of MASLD. Daily nutrition should prioritize products rich in anti-inflammatory compounds, substances that modulate lipid metabolism, and elements that support a healthy gut microbiota.

Key food products particularly recommended for their beneficial nutritional content include:

• A variety of vegetables and fruits
• Fermented products
• Whole-grain cereals
• Fish, eggs, legumes, white meat
• Plant-based oils
• Coffee

Among the numerous dietary patterns that counteract MASLD, the Mediterranean Diet is the most strongly recommended by experts as a dietary model for liver steatosis therapy. However, the wide array of dietary models and individual components with proven efficacy against MASLD allows for the personalization of dietary interventions to suit patients’ preferences and capabilities.

Long-term beneficial dietary behavior change and the incorporation of recommended components into daily nutrition can effectively counteract the development of MASLD while simultaneously promoting the patient’s overall metabolic health.

## Figures and Tables

**Table 1:** Dietary components counteracting MASLD: Mechanisms and nutritional sources.

Component	Mechanism	Daily Dosage	Dietary Sources	References
Vitamin E	Anti-inflammatory; improves liver histology; modulates liver enzymes	800 IU [15]	Vegetable oils, nuts, seeds, sprouts	[15,17,18]
Vitamin C	Antioxidant; prevents fibrosis	<30.9–≥67.0 umol/L [20]	Parsley, bell pepper, blackcurrant, kiwi	[16,19,20]
Coenzyme Q10	Anti-inflammatory; regulates lipid metabolism; reduces hepatic steatosis	240 mg [22]	Organ meats, meat, fatty fish	[21,22]
Polyphenols	Antioxidant and anti-inflammatory; inhibit hepatic fat accumulation; support lipolysis, lipophagy, and antifibrotic activity; improve lipid metabolism, gut barrier function, and liver enzyme profile	Quercetin 500–1000 mg Curcumin 80–3000 mg Silymarin 420 mg Ginger polyphenols 1500 mg [25,27,31,34]	Red grapes, pomegranate, blackberries, leafy greens, tomatoes, green tea, vegetable oils, strawberries, raspberries, ginger	[25,27,30,31,34]
Choline	Prevents liver fat accumulation; reduces risk of fibrosis	Men 550 mg Women 425 mg [37]	Egg yolks, organ meats, meat, milk	[35,37]
Alpha-lipoic acid	Anti-inflammatory; improves metabolic parameters	500 mg/kg body weight [39]	Organ meats, spinach, broccoli	[38,39,40]
Betaine	Strengthens gut barrier; antisteatotic; improves insulin sensitivity		Sugar beet, sprouts, whole grains	[41,42,43]
Berberine	Activates AMPK; improves gut barrier and insulin sensitivity; anti-inflammatory	500–1500 mg [44]	Barberry shrub	[44,45,46]
Fermented foods and probiotics	Modulate microbiota; improve gut barrier; anti-inflammatory; reduce steatosis and liver enzyme levels	Yogurt 300 g Kimchi 100 mg/kg [49,55]	Fermented dairy, pickled vegetables/fruits, kimchi, kombucha, sourdough, sauerkraut, beer, wine	[6,49,51,55,56]
Beta-glucans	Antisteatotic; anti-inflammatory; reduce risk of metabolic diseases	61.4 g/kg diet [57]	Whole grains, vegetables, fruits, legumes	[57,58]
Spirulina and chlorella	Anti-inflammatory; lower liver enzymes; enhance insulin action	20 g [60]	Microalgae (spirulina, chlorella)	[60,61,62]
Coffee	Anti-inflammatory; regulates lipid metabolism; inhibits steatosis and fibrosis progression	≥2 cups [67]	Coffee	[63,64,67]

## References

[1] Ambroselli D., Masciulli F., Romano E., Catanzaro G., Besharat Z.M., Massari M.C., Ferretti E., Migliaccio S., Izzo L., Ritieni A. (2023). New Advances in Metabolic Syndrome, from Prevention to Treatment: The Role of Diet and Food. Nutrients.

[2] Rappaport S.M. (2016). Genetic Factors Are Not the Major Causes of Chronic Diseases. PLoS ONE.

[3] Abdelhameed F., Kite C., Lagojda L., Dallaway A., Chatha K.K., Chaggar S.S., Dalamaga M., Kassi E., Kyrou I., Randeva H.S. (2024). Non-invasive Scores and Serum Biomarkers for Fatty Liver in the Era of Metabolic Dysfunction-associated Steatotic Liver Disease (MASLD): A Comprehensive Review From NAFLD to MAFLD and MASLD. Curr. Obes. Rep..

[4] Younossi Z.M., Kalligeros M., Henry L. (2025). Epidemiology of metabolic dysfunction-associated steatotic liver disease. Clin. Mol. Hepatol..

[5] Rinella M.E., Lazarus J.V., Ratziu V., Francque S.M., Sanyal A.J., Kanwal F., Romero D., Abdelmalek M.F., Anstee Q.M., Arab J.P. (2023). A multisociety Delphi consensus statement on new fatty liver disease nomenclature. Hepatology.

[6] Pouwels S., Sakran N., Graham Y., Leal A., Pintar T., Yang W., Kassir R., Singhal R., Mahawar K., Ramnarain D. (2022). Non-alcoholic fatty liver disease (NAFLD): A review of pathophysiology, clinical management and effects of weight loss. BMC Endocr. Disord..

[7] Ma Z., Hummel S.L., Sun N., Chen Y. (2021). From salt to hypertension, what is missed?. J. Clin. Hypertens. (Greenwich).

[8] Luna-Castillo K.P., Olivares-Ochoa X.C., Hernández-Ruiz R.G., Llamas-Covarrubias I.M., Rodríguez-Reyes S.C., Betancourt-Núñez A., Vizmanos B., Martínez-López E., Muñoz-Valle J.F., Márquez-Sandoval F. (2022). The Effect of Dietary Interventions on Hypertriglyceridemia: From Public Health to Molecular Nutrition Evidence. Nutrients.

[9] Feingold K.R., Ahmed S.F., Anawalt B., Blackman M.R., Boyce A., Chrousos G., Corpas E., de Herder W.W., Dhatariya K., Dungan K. (2024). The Effect of Diet on Cardiovascular Disease and Lipid and Lipoprotein Levels. Endotext [Internet].

[10] Ma X., Nan F., Liang H., Shu P., Fan X., Song X., Hou Y., Zhang D. (2022). Excessive intake of sugar: An accomplice of inflammation. Front. Immunol..

[11] European Association for the Study of the Liver (EASL), European Association for the Study of Diabetes (EASD), European Association for the Study of Obesity (EASO) (2024). EASL-EASD-EASO Clinical Practice Guidelines on the management of metabolic dysfunction-associated steatotic liver disease (MASLD). J. Hepatol..

[12] Rasheed Z., Rasheed N., Abdulmonem W.A., Khan M.I. (2019). MicroRNA-125b-5p regulates IL-1β induced inflammatory genes via targeting TRAF6-mediated MAPKs and NF-κB signaling in human osteoarthritic chondrocytes. Sci. Rep..

[13] Aghaei S.M., Hosseini S.M. (2024). Inflammation-related miRNAs in obesity, CVD, and NAFLD. Cytokine.

[14] Song Y., Ni W., Zheng M., Sheng H., Wang J., Xie S., Yang Y., Chi X., Chen J., He F. (2025). Chinese NAFLD Clinical Research Network (CNAFLD CRN). Vitamin E (300 mg) in the treatment of MASH: A multi-center, randomized, double-blind, placebo-controlled study. Cell Rep. Med..

[15] Higgins M.R., Izadi A., Kaviani M. (2020). Antioxidants and Exercise Performance: With a Focus on Vitamin E and C Supplementation. Int. J. Environ. Res. Public Health.

[16] Rychlik E., Stoś K., Woźniak A., Mojska H. (2024). Normy Żywienia Dla Populacji Polski.

[17] Chee N.M., Sinnanaidu R.P., Chan W.K. (2024). Vitamin E improves serum markers and histology in adults with metabolic dysfunction-associated steatotic liver disease: Systematic review and meta-analysis. J. Gastroenterol. Hepatol..

[18] Abera M., Suresh S.B., Malireddi A., Boddeti S., Noor K., Ansar M., Malasevskaia I. (2024). Vitamin E and Non-alcoholic Fatty Liver Disease: Investigating the Evidence Through a Systematic Review. Cureus.

[19] Zhao Y., Li H. (2022). Association of serum vitamin C with liver fibrosis in adults with nonalcoholic fatty liver disease. Scand. J. Gastroenterol..

[20] Xie Z.Q., Li H.X., Tan W.L., Yang L., Ma X.W., Li W.X., Wang Q.B., Shang C.Z., Chen Y.J. (2022). Association of Serum Vitamin C With NAFLD and MAFLD Among Adults in the United States. Front. Nutr..

[21] Gutierrez-Mariscal F.M., Arenas-de Larriva A.P., Limia-Perez L., Romero-Cabrera J.L., Yubero-Serrano E.M., López-Miranda J. (2020). Coenzyme Q_10_ Supplementation for the Reduction of Oxidative Stress: Clinical Implications in the Treatment of Chronic Diseases. Int. J. Mol. Sci..

[22] Vrentzos E., Ikonomidis I., Pavlidis G., Katogiannis K., Korakas E., Kountouri A., Pliouta L., Michalopoulou E., Pelekanou E., Boumpas D. (2024). Six-month supplementation with high dose coenzyme Q10 improves liver steatosis, endothelial, vascular and myocardial function in patients with metabolic-dysfunction associated steatotic liver disease: A randomized double-blind, placebo-controlled trial. Cardiovasc. Diabetol..

[23] Chen X., Chen B., Li Z., Ma L., Zhu Q., Liu C., He H., Zhang Z., Zhou C., Liu G. (2025). The Extract of Camellia Seed Cake Alleviates Metabolic Dysfunction-Associated Steatotic Liver Disease (MASLD) in Mice by Promoting Coenzyme Q Synthesis. Nutrients.

[24] Iqbal I., Wilairatana P., Saqib F., Nasir B., Wahid M., Latif M.F., Iqbal A., Naz R., Mubarak M.S. (2023). Plant Polyphenols and Their Potential Benefits on Cardiovascular Health: A Review. Molecules.

[25] Markowska J., Kasprzak-Drozd K., Niziński P., Dragan M., Kondracka A., Gondek E., Oniszczuk T., Oniszczuk A. (2024). Quercetin: A Promising Candidate for the Management of Metabolic Dysfunction-Associated Steatotic Liver Disease (MASLD). Molecules.

[26] Fang X., Song J., Zhou K., Zi X., Sun B., Bao H., Li L. (2023). Molecular Mechanism Pathways of Natural Compounds for the Treatment of Non-Alcoholic Fatty Liver Disease. Molecules.

[27] Huang Q., An Z., Xin X., Gou X., Tian X., Hu Y., Mei Z., Feng Q. (2024). The Effectiveness of Curcumin, Resveratrol, and Silymarin on MASLD: A Systematic Review and Meta-Analysis. Food Sci. Nutr..

[28] Hefer M., Petrovic A., Roguljic L.K., Kolaric T.O., Kizivat T., Wu C.H., Tabll A.A., Smolic R., Vcev A., Smolic M. (2024). Green Tea Polyphenol (-)-Epicatechin Pretreatment Mitigates Hepatic Steatosis in an In Vitro MASLD Model. Curr. Issues Mol. Biol..

[29] Zhang J., Wang S., Zhang T., Zi M., Wang S., Zhang Q. (2025). Green tea epigallocatechin gallate attenuate metabolic dysfunction-associated steatotic liver disease by regulation of pyroptosis. Lipids Health Dis..

[30] Hidalgo I., Ortiz-Flores M., Villarreal F., Fonseca-Coronado S., Ceballos G., Meaney E., Nájera N. (2022). Is it possible to treat nonalcoholic liver disease using a flavanol-based nutraceutical approach? Basic and clinical data. J. Basic Clin. Physiol. Pharmacol..

[31] Aghemo A., Alekseeva O.P., Angelico F., Bakulin I.G., Bakulina N.V., Bordin D., Bueverov A.O., Drapkina O.M., Gillessen A., Kagarmanova E.M. (2022). Role of silymarin as antioxidant in clinical management of chronic liver diseases: A narrative review. Ann. Med..

[32] Kim J.S., Song B.J., Cho Y.E. (2025). Pomegranate-Derived Exosome-Like Nanovesicles Containing Ellagic Acid Alleviate Gut Leakage and Liver Injury in MASLD. Food Sci. Nutr..

[33] Ghoreishi P.S., Shams M., Nimrouzi M., Zarshenas M.M., Lankarani K.B., Fallahzadeh Abarghooei E., Talebzadeh M., Hashempur M.H. (2024). The Effects of Ginger (*Zingiber officinale* Roscoe) on Non-Alcoholic Fatty Liver Disease in Patients with Type 2 Diabetes Mellitus: A Randomized Double-Blinded Placebo-Controlled Clinical Trial. J. Diet. Suppl..

[34] Samadi M., Moradinazar M., Khosravy T., Soleimani D., Jahangiri P., Kamari N. (2022). A systematic review and meta-analysis of preclinical and clinical studies on the efficacy of ginger for the treatment of fatty liver disease. Phytother. Res..

[35] Ishigure T., Sasase T., Tohma M., Uno K., Toriniwa Y., Saito T., Saigo Y., Edamura K., Miyajima K., Ohta T. (2023). Choline-deficient Diet-induced NAFLD Animal Model Recaptures Core Human Pathophysiology With Similar Gene Co-expression Networks. In Vivo.

[36] Chai C., Chen L., Deng M.G., Liang Y., Liu F., Nie J.Q. (2023). Dietary choline intake and non-alcoholic fatty liver disease (NAFLD) in U.S. adults: National Health and Nutrition Examination Survey (NHANES) 2017-2018. Eur. J. Clin. Nutr..

[37] DiStefano J.K. (2023). The Role of Choline, Soy Isoflavones, and Probiotics as Adjuvant Treatments in the Prevention and Management of NAFLD in Postmenopausal Women. Nutrients.

[38] Longhitano L., Tibullo D., Zuppelli T., Ronsisvalle S., La Spina E., Nicolosi A., Antoci M., Sipala F.M., Galvano F., Currenti W. (2024). (+)Alpha-Lipoic Acid Regulates Lipid Metabolism Gene Expression and Lipidic Profile in a Cellular Model of Fatty Acid Overload. Front. Biosci..

[39] Zwierz M., Chabowski A., Sztolsztener K. (2023). α-Lipoic acid—A promising agent for attenuating inflammation and preventing steatohepatitis in rats fed a high-fat diet. Arch. Biochem. Biophys..

[40] Cano Contreras A.D., Del Rocío Francisco M., Vargas Basurto J.L., Gonzalez-Gomez K.D., Amieva-Balmori M., Roesch Dietlen F., Remes-Troche J.M. (2025). Effect of alpha-lipoic acid and *Silybum marianum* supplementation with a Mediterranean diet on metabolic dysfunction-associated steatosis. World J. Hepatol..

[41] Perumal S.K., Arumugam M.K., Osna N.A., Rasineni K., Kharbanda K.K. (2025). Betaine regulates the gut-liver axis: A therapeutic approach for chronic liver diseases. Front. Nutr..

[42] Liu J., Liu Y., Chen Y., Liu Y., Huang C., Luo Y., Wang X. (2024). Betaine alleviates nonalcoholic fatty liver disease (NAFLD) via a manner involving BHMT/FTO/m^6^A/PGC1α signaling. J. Nutr. Biochem..

[43] Chen W., Xu M., Xu M., Wang Y., Zou Q., Xie S., Wang L. (2022). Effects of betaine on non-alcoholic liver disease. Nutr. Res. Rev..

[44] Vrentzos E., Pavlidis G., Korakas E., Kountouri A., Pliouta L., Dimitriadis G.D., Lambadiari V. (2025). Nutraceutical Strategies for Metabolic Dysfunction-Associated Steatotic Liver Disease (MASLD): A Path to Liver Health. Nutrients.

[45] Wang H., Zhang H., Gao Z., Zhang Q., Gu C. (2022). The mechanism of berberine alleviating metabolic disorder based on gut microbiome. Front. Cell. Infect. Microbiol..

[46] Ji J., Li Y., Xu T., Shao Q., Sun Z., Chen S., Zhang D., Wang Q., Wang X., Ma C. (2025). Protective effects of berberine on MASLD: Regulation of glucose and lipid metabolism through PI3K/Akt and STING pathways. Naunyn Schmiedebergs Arch. Pharmacol..

[47] Tilg H., Adolph T.E., Trauner M. (2022). Gut-liver axis: Pathophysiological concepts and clinical implications. Cell Metab..

[48] Benedé-Ubieto R., Cubero F.J., Nevzorova Y.A. (2024). Breaking the barriers: The role of gut homeostasis in Metabolic-Associated Steatotic Liver Disease (MASLD). Gut Microbes.

[49] Ebrahimi-Mousavi S., Alavian S.M., Sohrabpour A.A., Dashti F., Djafarian K., Esmaillzadeh A. (2022). The effect of daily consumption of probiotic yogurt on liver enzymes, steatosis and fibrosis in patients with nonalcoholic fatty liver disease (NAFLD): Study protocol for a randomized clinical trial. BMC Gastroenterol..

[50] Mojka K. (2014). Probiotics, prebiotics and synbiotics—Characteristics and functions. Probl. Hig. Epidemiol..

[51] Xu Y., Wang Y., Zhao Q., Chen B., Wang N., Zhang T., Jiang Y., Wu Y., He N., Zha G. (2024). Dairy products intake and prevalence, incidence, and recovery of non-alcoholic fatty liver disease in Chinese population. Hepatol. Int..

[52] Maslennikov R., Ivashkin V., Efremova I., Poluektova E., Shirokova E. (2021). Probiotics in hepatology: An update. World J. Hepatol..

[53] Paul A.K., Lim C.L., Apu M.A.I., Dolma K.G., Gupta M., de Lourdes Pereira M., Wilairatana P., Rahmatullah M., Wiart C., Nissapatorn V. (2023). Are Fermented Foods Effective against Inflammatory Diseases?. Int. J. Environ. Res. Public Health.

[54] Cywka Ł., Nowak A., Bogus Z.K., Nowak A., Baran N., Bielak A., Szwed W., Maksymowicz M., Machowiec P. (2023). Kombucha—Fermented tea rich in nutrients and its impact on health—Review. J. Educ. Health Sport.

[55] Yun Y.R., Lee J.E. (2024). Kimchi attenuates endoplasmic reticulum stress-induced hepatic steatosis in HepG2 cells and C57BL/6N mice. Nutr. Res..

[56] Moreira G.V., Araujo L.C.C., Murata G.M., Matos S.L., Carvalho C.R.O. (2022). Kombucha tea improves glucose tolerance and reduces hepatic steatosis in obese mice. Biomed. Pharmacother..

[57] Kei N., Wong V.W.S., Lauw S., You L., Cheung P.C.K. (2023). Utilization of Food-Derived β-Glucans to Prevent and Treat Non-Alcoholic Fatty Liver Disease (NAFLD). Foods.

[58] Jaeger J.W., Brandt A., Gui W., Yergaliyev T., Hernández-Arriaga A., Muthu M.M., Edlund K., Elashy A., Molinaro A., Möckel D. (2024). Microbiota modulation by dietary oat beta-glucan prevents steatotic liver disease progression. JHEP Rep..

[59] Służały P., Paśko P., Galanty A. (2024). Natural Products as Hepatoprotective Agents-A Comprehensive Review of Clinical Trials. Plants.

[60] Fakhoury-Sayegh N., Hamdan A., Lebbos S., Itani T., Trak-Smayra V., Khazzaka A., Dagher-Hamalian C., Sayegh L.N., Mallah M., Obeid O. (2024). Spirulina (*Arthrospira platensis*) Improved Nonalcoholic Fatty Liver Disease Characteristics and Microbiota and Did Not Affect Organ Fibrosis Induced by a Fructose-Enriched Diet in Wistar Male Rats. Nutrients.

[61] Yarmohammadi S., Hosseini-Ghatar R., Foshati S., Moradi M., Hemati N., Moradi S., Kermani M.A.H., Farzaei M.H., Khan H. (2021). Effect of *Chlorella vulgaris* on Liver Function Biomarkers: A Systematic Review and Meta-Analysis. Clin. Nutr. Res..

[62] Moradi M.N., Behrouj H., Alipoor B., Kheiripour N., Ghasemi H., Ghasemi H. (2021). Chlorella vulgaris is an effective supplement in counteracting non-alcoholic fatty liver disease-related complications through modulation of dyslipidemia, insulin resistance, and inflammatory pathways. J. Food Biochem..

[63] Arroyave-Ospina J.C., Martínez M., Buist-Homan M., Palasantzas V., Arrese M., Moshage H. (2025). Coffee Compounds Protection Against Lipotoxicity Is Associated with Lipid Droplet Formation and Antioxidant Response in Primary Rat Hepatocytes. Antioxidants.

[64] Ziółkiewicz A., Niziński P., Soja J., Oniszczuk T., Combrzyński M., Kondracka A., Oniszczuk A. (2024). Potential of Chlorogenic Acid in the Management of Metabolic Dysfunction-Associated Steatotic Liver Disease (MASLD): Animal Studies and Clinical Trials-A Narrative Review. Metabolites.

[65] Kaur M., Murugesan S., Singh S., Uy K.N., Kaur J., Mann N., Sekhon R.K. (2023). The Influence of Coffee on Reducing Metabolic Dysfunction-Associated Steatotic Liver Disease in Patients With Type 2 Diabetes: A Review. Cureus.

[66] Xin X., Chen C., Xu X., Lv S., Sun Q., An Z., Chen Y., Xiong Z., Hu Y., Feng Q. (2025). Caffeine ameliorates metabolic-associated steatohepatitis by rescuing hepatic Dusp9. Redox Biol..

[67] Lee J.H., Park J., Ahn S.B. (2023). Different Associations of Coffee Consumption with the Risk of Incident Metabolic Dysfunction-Associated Steatotic Liver Disease and Advanced Liver Fibrosis. Nutrients.

[68] Vahid F., Rahmani D., Hekmatdoost A. (2021). The association between dietary antioxidant index (DAI) and nonalcoholic fatty liver disease (NAFLD) onset; new findings from an incident case-control study. Clin. Nutr. ESPEN.

[69] Pourmontaseri H., Bazmi S., Sepehrinia M., Mostafavi A., Arefnezhad R., Homayounfar R., Vahid F. (2025). Exploring the application of dietary antioxidant index for disease risk assessment: A comprehensive review. Front. Nutr..

[70] Clemente-Suárez V.J., Mielgo-Ayuso J., Martín-Rodríguez A., Ramos-Campo D.J., Redondo-Flórez L., Tornero-Aguilera J.F. (2022). The Burden of Carbohydrates in Health and Disease. Nutrients.

[71] Czapla B.C., Dalvi A., Hu J., Moran I.J., Wijarnpreecha K., Chen V.L. (2025). Physical activity, diet, and social determinants of health associate with health related quality of life and fibrosis in MASLD. Sci. Rep..

[72] Jahromi M.K., Saber N., Norouzzadeh M., Daftari G., Pourhabibi-Zarandi F., Ahmadirad H., Farhadnejad H., Teymoori F., Salehi-Sahlabadi A., Mirmiran P. (2024). Carbohydrate quality index and risk of non-alcoholic fatty liver disease in Iranian adults. BMC Endocr. Disord..

[73] Paik J.M., Mir S., Alqahtani S.A., Younossi Y., Ong J.P., Younossi Z.M. (2022). Dietary Risks for Liver Mortality in NAFLD: Global Burden of Disease Data. Hepatol. Commun..

[74] Huang X., Gan D., Fan Y., Fu Q., He C., Liu W., Li F., Ma L., Wang M., Zhang W. (2024). The Associations between Healthy Eating Patterns and Risk of Metabolic Dysfunction-Associated Steatotic Liver Disease: A Case-Control Study. Nutrients.

[75] Guido D., Cerabino N., Di Chito M., Donghia R., Randazzo C., Bonfiglio C., Giannelli G., De Pergola G. (2024). A Dose-Response Study on the Relationship between White Meat Intake and Metabolic Dysfunction-Associated Steatotic Liver Disease (MASLD) in Southern Italy: Results from the Nutrihep Study. Nutrients.

[76] Donghia R., Tatoli R., Campanella A., Cuccaro F., Bonfiglio C., Giannelli G. (2024). Adding a Leafy Vegetable Fraction to Diets Decreases the Risk of Red Meat Mortality in MASLD Subjects: Results from the MICOL Cohort. Nutrients.

[77] Tan L.J., Shin S. (2022). Effects of oily fish and its fatty acid intake on non-alcoholic fatty liver disease development among South Korean adults. Front. Nutr..

[78] He K., Guo L.L., Tang H., Peng X., Li J., Feng S., Bie C., Chen W., Li Y., Wang M. (2022). A Freshwater Fish-Based Diet Alleviates Liver Steatosis by Modulating Gut Microbiota and Metabolites: A Clinical Randomized Controlled Trial in Chinese Participants With Nonalcoholic Fatty Liver Disease. Am. J. Gastroenterol..

[79] Chen X., Qiu W., Ma X., Ren L., Feng M., Hu S., Xue C., Chen R. (2024). Roles and Mechanisms of Choline Metabolism in Nonalcoholic Fatty Liver Disease and Cancers. Front. Biosci. (Landmark Ed.).

[80] Yiannakou I., Long M.T., Jacques P.F., Beiser A., Pickering R.T., Moore L.L. (2025). Eggs, Dietary Choline, and Nonalcoholic Fatty Liver Disease in the Framingham Heart Study. J. Nutr..

[81] Jiang Z., Kimura Y., Shirouchi B., Tanaka Y., Tsai W.T., Yuan X., Sato M. (2021). Dietary egg white protein hydrolysate improves orotic acid-induced fatty liver in rats by promoting hepatic phospholipid synthesis and microsomal triglyceride transfer protein expression. J. Nutr. Biochem..

[82] Tirosh O., Verman M., Ivancovsky-Wajcman D., Grinshpan L.S., Fliss-Isakov N., Webb M., Shibolet O., Kariv R., Zelber-Sagi S. (2024). Differential effects of low or high-fat dairy and fat derived from dairy products on MASLD. JHEP Rep..

[83] Langmann F., Ibsen D.B., Johnston L.W., Perez-Cornago A., Dahm C.C. (2025). Legumes as a Substitute for Red and Processed Meat, Poultry or Fish, and the Risk of Non-Alcoholic Fatty Liver Disease in a Large Cohort. J. Hum. Nutr. Diet..

[84] Yki-Järvinen H., Luukkonen P.K., Hodson L., Moore J.B. (2021). Dietary carbohydrates and fats in nonalcoholic fatty liver disease. Nat. Rev. Gastroenterol. Hepatol..

[85] Tedesco C.C., Bonfiglio C., Notarnicola M., Rendina M., Castellaneta A., Di Leo A., Giannelli G., Fontana L. (2023). High Extra Virgin Olive Oil Consumption Is Linked to a Lower Prevalence of NAFLD with a Prominent Effect in Obese Subjects: Results from the MICOL Study. Nutrients.

[86] Sokal-Dembowska A., Jarmakiewicz-Czaja S., Ferenc K., Filip R. (2024). Can Nutraceuticals Support the Treatment of MASLD/MASH, and thus Affect the Process of Liver Fibrosis?. Int. J. Mol. Sci..

[87] Jerab D., Blangero F., da Costa P.C.T., de Brito Alves J.L., Kefi R., Jamoussi H., Morio B., Eljaafari A. (2025). Beneficial Effects of Omega-3 Fatty Acids on Obesity and Related Metabolic and Chronic Inflammatory Diseases. Nutrients.

[88] Cansanção K., Citelli M., Leite N., López de Las Hazas M.C., Dávalos A. (2020). Tavares do Carmo MDG, Peres WAF. Impact of Long-Term Supplementation with Fish Oil in Individuals with Non-Alcoholic Fatty Liver Disease: A Double Blind Randomized Placebo Controlled Clinical Trial. Nutrients.

[89] Frankovic I., Djuricic I., Ninic A., Vekic J., Vorkapic T., Erceg S., Gojkovic T., Tomasevic R., Mamic M., Mitrovic M. (2024). Increased Odds of Metabolic Dysfunction-Associated Steatotic Liver Disease Are Linked to Reduced n-6, but Not n-3 Polyunsaturated Fatty Acids in Plasma. Biomolecules.

[90] Vell M.S., Creasy K.T., Scorletti E., Seeling K.S., Hehl L., Rendel M.D., Schneider K.M., Schneider C.V. (2023). Omega-3 intake is associated with liver disease protection. Front. Public Health.

[91] Commins I., Clayton-Chubb D., Fitzpatrick J.A., George E.S., Schneider H.G., Phyo A.Z.Z., Majeed A., Janko N., Vaughan N., Woods R.L. (2025). Associations Between MASLD, Ultra-Processed Food and a Mediterranean Dietary Pattern in Older Adults. Nutrients.

[92] Sun N., Prescott B., Ma J., Xanthakis V., Quatromoni P.A., Long M.T., Walker M.E. (2025). The cross-sectional association between ultra-processed food intake and metabolic dysfunction-associated steatotic liver disease. Clin. Nutr. ESPEN.

[93] Rinella M.E., Neuschwander-Tetri B.A., Siddiqui M.S., Abdelmalek M.F., Caldwell S., Barb D., Kleiner D.E., Loomba R. (2023). AASLD Practice Guidance on the clinical assessment and management of nonalcoholic fatty liver disease. Hepatology.

[94] Amamah S., Iatcu O.C., Covasa M. (2025). Dietary Influences on Gut Microbiota and Their Role in Metabolic Dysfunction-Associated Steatotic Liver Disease (MASLD). Nutrients.

[95] Liu J., Li C., Yang Y., Li J., Sun X., Zhang Y., Liu R., Chen F., Li X. (2025). Special Correlation between Diet and MASLD: Positive or Negative?. Cell Biosci..

[96] Steinberg G.R., Valvano C.M., De Nardo W., Watt M.J. (2025). Integrative Metabolism in MASLD and MASH: Pathophysiology and Emerging Mechanisms. J. Hepatol..

[97] Xiao M.-L., Lin J.-S., Li Y.-H., Liu M., Deng Y.-Y., Wang C.-Y., Chen Y.-M. (2020). Adherence to the Dietary Approaches to Stop Hypertension (DASH) Diet Is Associated with Lower Presence of Non-Alcoholic Fatty Liver Disease in Middle-Aged and Elderly Adults. Public Health Nutr..

[98] Del Bo’ C., Perna S., Allehdan S., Rafique A., Saad S., AlGhareeb F., Rondanelli M., Tayyem R.F., Marino M., Martini D. (2023). Does the Mediterranean Diet Have Any Effect on Lipid Profile, Central Obesity and Liver Enzymes in Non-Alcoholic Fatty Liver Disease (NAFLD) Subjects? A Systematic Review and Meta-Analysis of Randomized Control Trials. Nutrients.

[99] Sualeheen A., Tan S.-Y., Georgousopoulou E., Daly R.M., Tierney A.C., Roberts S.K., George E.S. (2024). Mediterranean Diet for the Management of Metabolic Dysfunction-Associated Steatotic Liver Disease in Non-Mediterranean, Western Countries: What’s Known and What’s Needed?. Nutr. Bull..

[100] Nilghaz M., Sadeghi A., Koochakpoor G., Poustchi H., Khodadadi N., Narimani B., Ghods M., Shafiee M., Shahparvari M.R., Hekmatdoost A. (2025). The Efficacy of DASH Combined with Time-Restricted Feeding (16/8) on Metabolic Associated Fatty Liver Disease Management: A Randomized Controlled Trial. Sci. Rep..

[101] Armandi A., Bugianesi E. (2024). Dietary and Pharmacological Treatment in Patients with Metabolic-Dysfunction Associated Steatotic Liver Disease. Eur. J. Intern. Med..

[102] Jia G., Jia M., Li C. (2024). The Moderating Effect of Dietary Fiber Intake on the Association between Sleep Pattern and Liver Fibrosis in Metabolic Dysfunction-Associated Steatotic Liver Disease: A Study from NHANES. BMC Gastroenterol..

[103] Castelnuovo G., Perez-Diaz-del-Campo N., Rosso C., Armandi A., Caviglia G.P., Bugianesi E. (2024). A Healthful Plant-Based Diet as an Alternative Dietary Approach in the Management of Metabolic Dysfunction-Associated Steatotic Liver Disease. Nutrients.

[104] Moss K., Gitman V., Pinto Sanchez M.I., Oczkowski S., Armstrong D., Jayakumar S., Karvellas C.J., Selzner N., Dionne J. (2024). Evidence Related to a Vegetarian Diet and Metabolic Dysfunction-Associated Steatotic Liver Disease: Protocol for a Scoping Review. BMJ Open.

[105] Quesada-Vázquez S., Aragonès G., Del Bas J.M., Escoté X. (2020). Diet, Gut Microbiota and Non-Alcoholic Fatty Liver Disease: Three Parts of the Same Axis. Cells.

[106] Faienza M.F., Cognetti E., Farella I., Antonioli A., Tini S., Antoniotti V., Prodam F. (2024). Dietary Fructose: From Uric Acid to a Metabolic Switch in Pediatric Metabolic Dysfunction-Associated Steatotic Liver Disease. Crit. Rev. Food Sci. Nutr..

[107] Faienza M.F., Baima J., Cecere V., Monteduro M., Farella I., Vitale R., Antoniotti V., Urbano F., Tini S., Lenzi F.R. (2025). Fructose Intake and Unhealthy Eating Habits Are Associated with MASLD in Pediatric Obesity: A Cross-Sectional Pilot Study. Nutrients.

[108] Kord-Varkaneh H., Salehi-Sahlabadi A., Tinsley G.M., Santos H.O., Hekmatdoost A. (2023). Effects of Time-Restricted Feeding (16/8) Combined with a Low-Sugar Diet on the Management of Non-Alcoholic Fatty Liver Disease: A Randomized Controlled Trial. Nutrition.

[109] Chirapongsathorn S., Rintaravitoon W., Tangjaturonrasme B., Chotsriluecha S., Pumsutas Y., Kanchanapradith A., Treeprasertsuk S. (2025). Effect of a Ketogenic Diet on Metabolic Dysfunction-Associated Steatotic Liver Disease (MASLD) Progression: A Randomized Controlled Trial. JGH Open.

